# Extracellular Vesicles in Transplantation

**DOI:** 10.3389/fimmu.2022.800018

**Published:** 2022-02-03

**Authors:** Nicolas Sailliet, Matti Ullah, Amandine Dupuy, Amanda K. A. Silva, Florence Gazeau, Hoa Le Mai, Sophie Brouard

**Affiliations:** ^1^ Nantes Université, INSERM, Centeer for Research in Transplantation and Translational Immunology, UMR 1064, Nantes, France; ^2^ MSC-med, INSERM U7057, Universite de Paris, Paris, France; ^3^ Labex IGO, Nantes, France

**Keywords:** extracellular vesicles, biomarkers, transplantation, immunomodulation, immunology

## Abstract

Extracellular vesicles (EVs) have been extensively studied in the last two decades. It is now well documented that they can actively participate in the activation or regulation of immune system functions through different mechanisms, the most studied of which include protein–protein interactions and miRNA transfers. The functional diversity of EV-secreting cells makes EVs potential targets for immunotherapies through immune cell-derived EV functions. They are also a potential source of biomarkers of graft rejection through donor cells or graft environment-derived EV content modification. This review focuses on preclinical studies that describe the role of EVs from different cell types in immune suppression and graft tolerance and on the search for biomarkers of rejection.

## Introduction

Communication among cells is an essential event in all living organisms that is achieved through several mechanisms, among which secretion of soluble elements is an important factor. For the past two decades, a new method of intercellular communication has been unleashed through secretion of membrane-bound, nanosized particles known as extracellular vesicles (EVs). Briefly, EVs are lipid bilayer vesicles with a size range of 50–2,000 nm in diameter that are released by cells into the extracellular spaces and are recognized as a new method of intracellular communication with a range of signaling (1, 2). They are considered cargo delivery vesicles because they harbor nucleic acids, proteins, lipids and metabolites that reflect their cellular origin. EVs are released in extracellular spaces during physiological and pathological conditions by several types of cells (3, 4). EVs are found in almost every fluid of the body from synovial fluids and breast milk to saliva, plasma and urine (2) and play fundamental roles in regulating normal physiological processes and in the progression of disease and reflect the state of parent cells at the time of release (5).

The term “extracellular vesicle” was first used in the literature in 1971 by a study that involved electron microscopic analysis of the freshwater alga ‘Ochromonas danica’ (6). Stahl and Johnstone independently investigated and provided insights into the mechanisms involved in the secretion of EVs from reticulocytes (7, 8). At that time, several names were used to mention EVs, such as shedding vesicles, membrane fragments, plasma membrane vesicles, microvesicles and exosomes. One of the breakthroughs for EVs was in 1996, when Graca Raposo et al. reported the participation of EVs in immune responses by their role in activating the adaptive immune response (9).

## The Different Types of EVs

Extracellular vesicles can be classified on the basis of their biogenesis, origin, biological function or cargo (10). However, the most acceptable classification for EVs is based on their genesis. Generally, they are three classes of EVs known as exosomes, microvesicles and apoptotic bodies. The common term ectosomes can be used for MVs and apoptotic bodies, as both are shed through blubbing of the cellular membrane (11). In all three subtypes of EVs, there is a lipid bilayer membrane that surrounds their content, e.g., proteins, RNA, or cellular debris. Their genesis involves different mechanisms and has different sizes and buoyant densities (12). Apoptotic bodies are generally the largest in; however, their size ranges from 50 nm to 5000 nm, and their density ranges from 1.16–1.28. As indicated by their name, these vesicles are released by apoptotic cells containing some apoptotic bodies. However, in contrast to what their name suggests, they are not simple debris of apoptotic cells; these EVs have important roles in immune regulation and activate pathways to aid in phagocytosis and removal of dead cells (13).

The other two types of EVs, exosomes and microvesicles, are regularly secreted by cells to maintain intercellular communication. They are quite similar in properties and are very difficult to separate. The two have been characterized based on their size, morphology, and lipid bilayer composition (14). One of the major differences between the two is the way they are produced. Exosomes are formed through the endolysosomal pathway following invagination of endosomal membranes to form multivesicular bodies and are released after fusion of multivesicular bodies with the plasma membrane (15). Microvesicles are formed by outward budding of the membrane; they are larger in size ranging from 100 nm to 1000 nm, while exosomes are smaller in size ranging from 50 nm to 150 nm and have a buoyant density from 1.10 to 1.14 gram/ml. As exosomes are formed inside the cellular membrane, they are more enriched in phosphatidylserine, whereas the composition of microvesicles is closer to that of the parent cell (14). The general composition of both EVs is same, i.e., each contain cytoplasmic proteins, lipids, mRNA, miRNA and receptors. Some criteria identified in the Minimal information for studies of extracellular vesicles MISEV 2018 attempt to better discriminate exosome-specific features. However, considering the difficulties in determining the precise classification, the terminology extracellular vesicles is recommended as a more appropriate designation.

In addition to the abovementioned classification, EVs can be classified to reflect their tissue origin. Prostasomes are EVs that originate from the prostate, while epididymosomes are vesicles secreted by epididymal epithelial cells. However, these terms include all the EVs that have the same origin irrelevant to the type of EVs present or the way they are produced. Hence, there is confusion in identifying the true nature of EVs. Other examples of such classification include cardiosomes (heart), dexosomes (dendritic cells), oncosomes/texosomes (tumor cells) and others (16).

## The Cargo Components and Functions of EVs

EVs are membrane-bound vesicles that reflect the composition of the cells from which they are derived. Hence, depending on the origin, EVs contain different types of proteins, mRNAs, miRNAs, DNA and lipids (including cholesterol and receptors, cytokines and low molecular weight metabolites, e.g., glutathione, amino acids and ATP).

As found in most of the fluids in the body, EVs are associated with regular physiological processes, especially those that involve communication between cells (17) such as stem cell pluripotency (18), angiogenesis (19–21), coagulation (22, 23) and either immune activation or attenuation (14, 24, 25). EVs also impact various pathological conditions, among which the most documented is cancer formation through angiogenesis or cell-cell communication (22, 26). EVs favor HIV-1 infection through the transfer of chemokine receptor 5, facilitating viral entry, and neurodegenerative diseases such as Alzheimer’s disease, where EVs are associated with amyloid β peptides, help in the deposition of this toxic protein to several parts of the brain (27, 28). EVs function through the cargo they carry to specific delivery sites. Either through direct action, such as matrix degradation by MMP carried by EVs that aid in the formation of capillary structures (20), or through the activation/inhibition of “resident” cells, EVs can stimulate the production of ROS that decrease NO and inhibit the VEGF-induced pathway (21). The functions of EVs can vary based on the type of components they carry. They may be immune suppressive or immune stimulatory depending on the various components present in EVs.

### Proteins

The protein composition of EVs has been extensively studied through different techniques and coined in different databases, e.g., Urinary Exosomes Protein Database (http://dir.nhlbi.nih.gov/papers/lkem/exosome/) and ExoCarta (http://exocarta.ludwig.edu.au/). This component is essentially a characteristic of the origin cells. Lötvall et al. categorized proteins present in EVs into four distinct groups. Most EVs contain a common group of proteins that are involved in vesicle structure, genesis and transport and are known as vesicular proteins. These proteins are transmembranous or lipid bound and mainly include tetraspanins (CD9, CD63 and CD81), integrins that help attachment EVs, and other cell adhesion molecules (such as PECAM-1, ICAM-1 and VCAM-1). The second group of proteins is those present in the cytosol that can bind to the membrane or receptors, e.g., endosome-binding proteins (annexins) and signal transduction proteins (syntenin). The other groups of proteins are intracellular proteins that are present in different compartments, such as mitochondrial proteins (cytochrome C) and Golgi components (GM130). Finally, the last group is composed of extracellular proteins that are membrane bound, e.g., acetylcholinesterase, ECM (including fibronectin and collagen type IV) and other soluble proteins, such as cytokines (bFGF, VEGFR2, TGF-β) and growth factors (29, 30).

### Nucleic Acids

EVs contain different nucleic acids, such as mRNA, miRNA, long noncoding RNAs, rRNA, tRNA mitochondrial DNAs and short DNA sequences (31), collected in the Exocarta database. mRNA or miRNAs can be transferred functionally to other cells, resulting in reprogramming of recipient cells (18, 32, 33). Different studies have shown that mRNA in EVs is functional and can be translated to proteins in recipient cells (34, 35). miRNAs have been extensively studied in the context of cancer, and several miRNAs have been identified to be linked to immune suppression that promotes cancer growth or is associated with other diseases, such as Alzheimer’s disease (36–38). The treatment of MSCs with high concentrations of RNAse significantly decreased the protective effect of MSC-derived EVs in ischemia-reperfusion-induced kidney patients, suggesting an important role of exosomal RNAs in epigenetic reprogramming necessary for the regenerative effect of MSC-derived EVs (39). Additionally, the presence of miRNAs in EVs has been linked to enhanced engraftment and hematopoietic stem cell function upon treatment (40).

### Lipids

Extracellular vesicles have lipids as an essential component of their structure that maintain vesicular integrity, rigidity, function and intercellular fusion of EVs. Some of the lipids are more enriched in EVs than in their cells of origin. Although the proportion of different types of lipids varies from cell to cell, cholesterol is the major form of EV present in all sources, ranging from 15% (mast cells) to 59% (PC-3 cells; prostate cancer cell line) (41, 42). Other lipid constituents are sphingomyelin, phosphatidylcholine, phosphatidylserine, ganglioside GM3, lysophospholipids and phosphatidylinositol. EVs also contain prostaglandins such as PGE2, PGF2, PGJ2 and PGD2 at a concentration that is enough to trigger biological responses such as PPARγ or LxR pathways in target cells, which can further suppress the transcription of proinflammatory cytokine and chemokines mRNA (43, 44).

## EV-Induced Immune Modulation in the Management of Solid Organ Transplantation

Organ replacement is the best option for several organ failure diseases in terms of quality of life and survivability. Through kidney, heart, lung, liver and pancreas transplantation, 2.3 million life-years were gained in 2017 in the US. Nevertheless, the risk of rejection is a major concern with a poor long-term outcome due to chronic rejection even with immunosuppressive treatments. The lack of donors makes transplantation a scarce resource. Thus, it is important to develop new strategies to improve graft survival. EVs can either activate or suppress the immune system depending on the type of cargo and the cells from which they are produced. For example, EVs produced by T cells help prime DCs through the transfer of DNA in a feedback mechanism, while EVs produced by tumor cells are enriched in several miRNAs, such as miR-21–3-p and miR-181d-5p, that can reprogram neighboring macrophages to support tumor growth by suppressing the immune system (45, 46). Whether EVs stimulate or suppress the immune system depends on EV-secreting cells. For example, an *in vivo* study showed that the administration of exosomes derived from mature or immature DCs had different protein compositions. Mature DC exosome administration resulted in induced effector T cells that led to skin graft rejection, while exosomes derived from immature DCs resulted in T cell activation but not skin graft rejection. This was linked to the absence of ICAM-1 and MHC-II molecules on exosomes derived from immature DCs (47).

The role of EVs in the induction of graft rejection has been extensively studied. It is now well established that the direct recognition of donor cells is not the major transplant recognition pathway. EVs carrying donor MHC molecules and peptides can initiate the immune response that ultimately results in graft rejection. Marino et al. showed that although donor dendritic cells still reside in donor tissue, host APCs in lymph nodes present vesicles bearing donor MHC I and II molecules and are responsible for T cell activation after skin and heart transplants (48). This suggests that host APCs can acquire donor MHC molecules present on EVs secreted by donor cells and thereby initiate immune responses and further determine the fate of the allograft in a semi-direct pathway (24). In another study, Becker et al., described the role of EVs in allograft recognition in an indirect pathway (49). The semi-direct pathway have been linked with the cross dressing of donor class I MHC onto host APC that favors the “three cell model” of alloreactive CD8+ T cell activation (50). Moreover, in the model of fetal tolerance to maternal antigens during pregnancy proposed by W.Burlingham in which maternal (or donor) cells generates MHC-containing exosomes leading to semi-direct and indirect pathways, the indirect pathway does induce tolerogenic APC through the co-expression of PD-L1 and CD86 with donor MHC. Whereas in the semi-direct pathway, the APC side of the immune synapse is exclusively composed of donor exosomal material where PD-L1 and CD86 are absent and thus, induces clonal activation of CD4 T cells (51). As a result, EVs are a major interface between donor and host environment and this suggest a potential therapeutic use of donor EVs to promote tolerance. This would require the inhibition of the semi-direct pathway in those APC to prevent effector T cell activation. This has not been reported in the literature which focuses on the use of EVs that have been shown to regulate physiological processes linked to graft survival improvement such as immune modulation (52) and tissue repair (53). There are several studies testing the effects of EVs in the prevention of allograft rejection (see parts 2.1-2.5). We still need to know which one to use. Although EVs can be secreted by virtually all cell types, it is still unclear which cell types can produce the EVs that will efficiently prevent graft rejection or induce graft tolerance. The outcome will be highly dependent of the producing cell type and the presence of donor MHC molecules (54). We herein review the most studied candidates for EV therapies: Mesenchymal stromal cells (MSC)-EVs, DC-EVs and Tregs-EVs. Other cell types produce EVs with immunoregulatory properties: Platelet-EVs or neutrophils-EVs but for now, no data are available in transplantation models. Thus, these EVs are only mentioned.

### MSC-Derived EVs

MSCs are nonimmune cells with immunoregulatory properties capable of inhibiting immune effector cell activity (55) and are thus a suitable candidate for EV therapies. MSC-derived EVs exert their protective effects on allografts through the modulation of different biological processes, such as inflammation (56), apoptosis (57), fibrosis (58), angiogenesis (59) and tissue repair (60). When cocultured with CD2/CD3/CD28-stimulated T cells or PHA-stimulated PBMCs, human MSC-derived EVs were able to decrease the proliferation rate, maturation and migration ability of T cells, B cells and NK cells (61) and to reduce IFN-γ production by T cells (62). In a chronic GvHD mouse model, treatment with MSC-derived EVs reduced the percentage of T follicular helper, germinal center B cells and macrophages as well as macrophage production of TGFβ and SMAD2 in the skin (63). In a rat model of kidney allografts, adipose tissue-derived MSC-EVs reduced T and NK cell graft infiltration but did not prolong graft survival, whereas bone marrow MSC-derived EVs from donors slightly improved graft survival but did not affect graft lymphocyte infiltration (54). This study and others suggests that the origin of EVs may be determinant in the development of EV-based therapies, as MSC-derived EVs have different immunoregulatory and regenerative functions depending on their origin (64). Interestingly, bone marrow MSC-derived EVs from chronic kidney disease (CKD) patients showed no differences from those from healthy donors in the capacity to induce angiogenesis and tissue repair, as measured by *in vitro* assays (65), suggesting that bone marrow MSC-derived EVs are fully functional in CKD patients.

In a mouse model of renal ischemia/reperfusion Injury (IRI), administration of bone marrow-derived MSC-EVs reduced renal epithelial cell apoptosis and improved kidney function (66). These results are in accordance with the results from other teams working on other types of MSC-EVs, including Wharton’s jelly MSC-EVs (67), human induced pluripotent stem cells derived MSC-EVs (68), and BM MSC-EVs (39) using different models such as in hepatic (69, 70) lung (71, 72), islets (73), BM (63) or heart (67) transplantation models.

MSC-EV injection also has a protective role when perfused *ex vivo* in lungs from deceased donors that did not match the criteria for transplantation. This was associated with an elevation in the rate of alveolar fluid clearance. These data suggest that EV injections before surgery could be part of the treatments and could increase the potential donor pool (74). Interestingly, administration of bone marrow MSC-derived EVs to a patient with GVHD decreased proinflammatory cytokine responses and improved clinical symptoms (75).

### DC-Derived EVs

Exosomes derived from mature DCs or immature DCs have different protein compositions. *In vivo*, mature DC exosome administration resulted in induced effector T cells that led to skin graft rejection. However, exosomes derived from immature DCs resulted in T cell activation without rejection (47).

Immature DC exosomes (imDEX) express low levels of ICAM-I, MHC-II, CD80 and CD86, which characterize suppressive functions (47). Four studies described the role of donor-derived imDEX in mouse and rat transplantation models (76–79) in kidney, liver, heart and intestinal transplantation. Li et al. showed in a cardiac transplant model in BALB-C mice that donor-derived imDEX prolong graft survival. These EVs induce IL-10, inhibit IFN-γ and IL-17 mRNA production by T-cells and favor FoxP3 expression in the T-cell compartment. Together, these data corroborate the induction of tolerance by imDEX. In these studies, EVs promoted short-term graft survival. The best results were obtained when EVs were injected in combination with either rapamycin or Tregs. However, imDEX has not been studied in chronic allograft models.

### Treg-Derived EVs

In a rat kidney transplantation model, EVs secreted by regulatory T-cells are immunosuppressive in nature and inhibit T-cell proliferation (52). Murine Tregs secrete more EVs than other murine T cells, such as CD4^+^, CD8^+^ and Th1 cells (80). Moreover, in a mouse model, EVs secreted by allo-Tregs are known to have specific miRNAs. miR-150–5p and miR-142–3p suppress IFN-γ secretion by T helper cells (81) and IL-6 secretion by DCs while increasing anti-inflammatory IL-10 production by DCs (82). miR-Let7d reduces Th1 cell proliferation and cytokine production (TNF, IFN-γ) (83). EVs derived from Tregs reduce CD4+ T cell proliferation and decrease proinflammatory cytokine release, e.g., IL-2 and IFN-γ. This has been attributed to the presence of the ectoenzyme NT5E or CD73, which plays a role in the immunosuppressive function of Tregs by binding to the adenosine receptor A2aR (84–87). This tolerogenic profile prolongs renal allograft survival in a model of acute rejection in rats and inhibits effector T-cell proliferation (88). Treg-EVs also decrease CD8+ cytotoxic T lymphocyte (CTL) proliferation and viability. Interestingly, the proportion of CTLs in G0/G1 was higher when cocultured with Treg-EVs than when cocultured with Tregs alone. Alongside CTL proliferation, Treg-EVs decrease perforin and IFN-γ production. When treated with Treg-EVs, liver-transplanted rats had better short-term survival (89).

### Macrophage-Derived EVs

Macrophages, which are mainly part of innate immunity but also contribute to adaptive immunity (90), release EVs that carry pathogen-associated molecular pattern (PAMP) or invader (e.g., mycobacteria) components that result in increased cytokine production by macrophages *via* Toll-like receptors (TLRs) and by memory CD4^+^ and CD8^+^ T cells (91, 92). Macrophage-derived EVs contain miR-223, which helps differentiate myeloid cells and plays a role in graft rejection (93, 94). Macrophage-derived EVs present enzymes for leukotriene biosynthesis, suggesting the ability to induce *in vitro* neutrophil chemotaxis (95, 96). The stimulation of macrophages with LPS causes secretion of EVs enriched in cytokines and miRNA, which promote inflammation through activation of NF-κB pathways in naive immune cells (97). Furthermore, EVs can be identified as M1 or M2-like depending on the type of macrophages that secrete the EVs (98), with EVs from M2 macrophages that help promote gastric cancer cell migration. This suggests a role of these EVs either in supporting cancer cells or in immune suppression (99).

### Modified EVs

*In vitro* modifications of EVs may improve their therapeutic potential, either to increase their suppressive function or to deliver drugs. Engineering the parent cell is an interesting way to modify EVs without *in vitro* manipulation of the EVs themselves. However, Tapparo et al. reported that EVs enriched with different miRNAs, known to be pro-regenerative, were ineffective in ameliorating graft fate compared to naive EVs (100). In their study, they only tested 3 miRNAs, miR-127 and miR-10a miR-486. Further studies may highlight other miRNAs or proteins whose enrichment would be beneficial.

EV modifications are not necessarily directed toward the immunosuppressive activity of EVs. To be effective, EVs delivery needs to be directed toward the graft environment, and EVs elimination may be reduced. In this aim, scaffolds were engineered to promote EVs deposition in the graft. For example, a MMP2-sensitive self-assembling antigen (KMP2) hydrogel releases EVs only in the presence of metalloproteases and other endogenous proteases and peptidases. KMP2-EVs were shown to have greater bioactivity in a renal IRI model than EVs alone (101). In another example, to increase EV bioavailability, EVs may be coated with the hydrophobic polymer PEG to reduce liver uptake (102) and then functionalized with cell-specific nanobodies (103). Modified EVs may also be synthetized *in vitro*, out of the cell, to provide continuous delivery of pro-tolerogenic factors (104). Such microparticles delivering cytokines (TGFβ1 and IL-2) or drugs (rapamycin) were reported to induce Tregs and promote allograft survival in animal models (105).

## EVs as a Source of Biomarkers of Graft Rejection

In addition to expectancies as therapeutics, EVs carry a fraction of their parent cell intracellular and membrane proteins. Making it one more source of biomarkers. The advantage of EVs as biomarkers compared to engrafted cells is their circulating ability through different fluids, the most obvious being serum, which makes EV harvesting less invasive than biopsies. There is no consensus method for EV isolation and the duration of the procedures as well as their cost are variable. EVs purity and integrity after the procedure is also an important point that has to be considered. Differential ultracentrifugation is the most used method for EVs purification. It is easy-to-use but the procedure is long and require specific equipment. Ultrafiltration and PolyEthyleneGlycol-Based precipitation are fast, low-cost and easy-to-use protocols counterbalanced by relatively important contaminations with low- mRNA/miRNA quality. Other methods, based on immunoaffinity of tetraspanin detection such as CD9 expression allow better purity, yet may exclude CD9neg EVs. Moreover, the removal of antibodies uses harsh protocols that may alter membrane-bound proteins. Finally, the size exclusion chromatography has demonstrated good purification performances. Yet, better purifications require more expensive columns. All these methods strengths and weaknesses are more extensively discussed here (106).

### Biomarkers of Renal Transplant Rejection in Blood and Urine

Kidney rejection is associated with different pathological conditions that induce changes in the urinary compartment that may also contain EVs. In humans, urine EVs are thus increased after transplantation and in chronic kidney diseases (107) and are a potential source of biomarkers (108). Ultracentrifugation isolated-EVs from blood of kidney transplant recipients with glomerulopathy have higher levels (up to 2-fold) of fibronectin and type IV collagen than EVs from patients with a stable transplant (109). These two molecules act as self-antigens and activate autoimmunity. Proteomics by mass spectrometry techniques on EVs isolated by differential centrifugations can discriminate cell-mediated rejection (CMR), antibody-mediated rejection (ABMR) and tubular injury (TI). Each of these pathways can be associated with biological processes: tubular injury is associated with elevated sodium ion transport, CMR with cytoskeletal organization and epithelial cell differentiation and ABMR with protein trafficking, inflammatory and complement pathways. The GO term “immune response” was enriched in the ABMR and CMR groups but not in the TI group (110). Urinary EVs from a long-term graft survival (LGS) group were compared with EVs from a chronic antibody-mediated rejection (CAMR) group through another proteomic analysis using nano-UPLC–MS/MS (Nanoscale liquid chromatography coupled to tandem mass spectrometry). Six proteins (known to be present in EVs and mentioned in the literature to be related to graft rejection) were upregulated in the CAMR group (APOA1, PIGR, TTR, AZGP1, HPX and CP). APOA1 was the most efficient biomarker in the list to predict graft outcome (111). The same analysis in urine from kidney transplanted patients with stable function and acute T cell-mediated rejection highlighted HPX and TSPAN1, and the combination of these markers can discriminate stable and acute TCMR (112). In another study, by using mass spectrometry of EVs content after differential centrifugations Sigdel et al. identified 11 proteins upregulated in EVs in urine from patients with acute rejection (A2M, APOA2, APOM, CD5L, CLCA1, FGA, FGB, IGHM, DEFA5, PROS1 and KIAA0753) (113).

### Biomarkers of Lung Rejection in Blood and Bronchoalveolar Lavages

EVs derived from the sera and bronchoalveolar lavage (BAL) fluids of patients with bronchiolitis obliterans syndrome (BOS), acute rejection (AR) and purified with ultracentrifugation have been described to contain the self-antigens Collagen-V and Kα1T. In the patients tested, an increase in collagen-V in EVs was visible as early as 1-month post transplantation, whereas the clinical diagnosis of acute rejection was at 1.5, 1.9 and 2.8 months. In contrast, collagen-V expression in EVs remained stable at 1 month and was undetectable at 3 and 6 months in stable patients. miRNA profiling in EVs from a cohort of 30 patients showed high expression of miR-92a and miR-182 involved in endothelial activation and inflammation in AR and BOS. miR-155 (inflammation) and miR-142–5p (antibody-mediated rejection) were linked to EVs from patients with BOS (114). In the same patients, these markers of rejection were also differentially expressed in EVs from BAL. Interestingly, Gunasekaran et al. showed that only EVs from patients rejecting their graft expressed the costimulatory molecules CD40, CD80 and CD86 (115). In another study, EVs in BAL fluids from patients with acute rejection overexpressed olfactory receptors, complement, surfactants, defensins, TLR2, MYD88, and nitric oxide synthase pathways and downregulated CXCL16, IL-33 and EEA-1 compared with EVs from BAL of patients with stable graft function (116).

### Biomarkers of Heart Rejection

Heart rejection has also been associated with changes in EVs content compared to patients stable patients. Acute Cellular Rejection (ACR) and ABMR were associated with significantly higher expression of the immune and nonimmune markers HLA-I, CD41b, ROR1, and SSEA-4 in EVs isolated by ultracentrifugation. ACR was specifically associated with the T-cell markers CD2 and CD3, and ABMR was associated with the B-cell markers CD19, CD20, HLA-II, CD25, and the epithelial cell adhesion molecule CD326 (117). Together, these markers have good potential for rejection diagnosis. When comparing composition of EVs isolated with a commercially available kit based on EV precipitation in heart transplanted patients, heart failure patients and healthy controls, there are greater similarities between healthy controls and heart failure patients than with transplanted patients with no rejection (118). This indicates the ability of EVs to describe the immune and nonimmune changes following transplantation independently of the rejection.

### Biomarkers of Islets Rejection

In islet transplantation, there were changes as early as day 1 in islet-derived EVs and T cell-derived EVs, where glucose kinetic changes were observed at day 6. Serum EV modifications are visible both in quantification (relative decrease in total serum EVs) and composition with the downregulation of heat shock cognate protein 70 (HSC70) and angiopoietin-1 and the upregulation of hemopexin, complement C3 and 39 miRNAs (2-fold increase or more). In a clinical cohort of islet-transplanted diabetic patients, the drop in islet-derived exosomes correlated with the rise in donor-specific antibodies, while clinical markers of rejection were still unchanged (119).

### Clinical Applications: Benefits and Limitations

EVs are a source of therapeutic and diagnostic tools that have not yet been fully explored and offer promising perspectives in the field of solid organ transplantation. Their use, like cellular therapies and other synthetic delivery vehicles (liposomes, nanoparticles), has many advantages. Due to their natural origin, EVs present better biocompatibility and biodistribution (120). Their membrane composed of a lipidic bilayer protects their molecular content and ensures their transport within fluids by protecting them from degradation (18, 121, 122). Moreover, their size has proven to be a major asset, allowing better accessibility in highly vascularized organs. Indeed, cell therapies, based for example on the use of MSCs, have shown that their invasive size (20-30µm) leads to their accumulation in pulmonary capillaries (5-10µm) when administered systemically (123). Finally, compared to liposomal or cellular therapies, EVs present a reduced toxicity and immunogenicity (124–126). In addition to the advantages linked to their intrinsic properties, EVs present a reduced and more adapted production time in comparison of cells therapy, as well as a capacity to be stored (127, 128). The production and use of EVs in an allogeneic production and administration model is of great interest, especially in the context of pathologies with a reduced window of therapeutic action (129).

Nevertheless, the use of EVs, whether for therapeutic or diagnostic purposes, also has a number of limitations. The absence of a consensus method for the isolation, purification and characterization of EVs makes their use in the clinic complex at the present time (130). Indeed, the current isolation methods do not allow a complete purification of EVs with a practically systematic co-isolation of proteins and contaminating particles of the same size/density or a yield in EVs that is too low (131). Moreover, the absence of markers results in a heterogeneous suspension of EVs, whose different subtypes and functionality remain difficult to determine (132). Furthermore, comparative studies agree that the use of different isolation and characterization techniques leads to heterogeneity of results obtained for the same samples (133). The development of diagnostic tools would then be subject to a material investment with a consequent cost, making the use and generalization of EVs as biomarkers complex. Finally, the content and properties of EVs remain dependent on the cell culture conditions. Hypoxia (134), serum deprivation (135), etc. are some of the factors that can modify the secretome of candidate cells, and consequently, their potential therapeutic activity. Obtaining stable batches of EVs of acceptable quality for clinical use will require further development to ensure the quality and safety of the EVs produced.

In addition to the technical aspects, the use of EVs is also subject to their biological source. Indeed, the production of EVs is conditioned by the availability of a sufficient quantity of cells, and the capacity to produce them in large quantities without altering their phenotype, morphology and content. In order to overcome this problem, several teams try to increase the secretion of EVs, not without consequences. The processes used generate cellular stress and impact the phenotype of the exposed cells. This leads to a modification of their secretory profile and the immunoregulatory properties of the generated EVs (136, 137).

It is undeniable that research is still required for the use of EVs in the clinic. Nevertheless, EVs could represent a new, more efficient and reliable therapeutic approach in solid organ transplantation, to complement and perhaps one day replace current treatments, whose side effects remain heavy and which are no longer sufficient for long-term graft stability.

## Conclusion

The acquisition of tolerance toward the graft is the final step to improve patient’s quality of life after transplantation. However, understanding the mechanisms of tolerance may require years/decades of research. Therefore, the management of transplantation is still dependent on two conditions: an adequate immune suppression to delay graft rejection and a continuous monitoring of graft rejection through different biomarkers. The use of EVs is a promising method for transplantation management. On the one hand, they have shown their ability to reflect the transplanted environment and to discriminate rejection events from stable patients through a rapid increase in immune-associated proteins and miRNA (summarized in **Table 1**). These changes are certainly linked to a function of the EV compartment in the process of graft rejection. EVs are a potent intermediate that promotes contact between donor antigens and the host immune system, as they are enriched in MHC and costimulatory molecules for most types of transplant rejection (139, 141–143).

**Table 1:** Markers of graft rejection in the EV compartment.

Transplanted organ	Syndrome	Origin of Evs	Biomarkers positively correlated with rejection	Biomarkers negatively correlated with rejection	Ref	Transplanted organ	Syndrome	Origin of Evs	Biomarkers positively correlated with rejection	Biomarkers negatively correlated with rejection	Ref
Lung	Bronchiolitis Obliterans Syndrome (BOS)	Isolated from BAL fluids and serum by ultracentrifugation	Collagen-V, Kα1T, miR-92a, miR-182, miR-155, miR-142-5p, olfactory receptors, complement, surfactants, defensins, TLR2, MYD88	CXCL16, IL-33, EEA-1	(114–116, 139)	Kidney	Kidney glomerulopathy	exosomes isolated from plasma by ultracentrifugation followed by sucrose cushion	Fibronectin, Collagen type IV		(109)
	Acute Rejection	Isolated from BAL fluids and serum by ultracentrifugation	miR-182, miR-92a		(114)		Tubular injury	Isolated from 20mL urines by differential centrifugation	sodium-ion transport (26 proteins)		(110)
Heart	Acute Cellular Rejection (ACR)	Isolated from plasma by differential centrifugation	KV302, HV304, ITIH1, FRMPD1	FIBB, FIBG, C1QA, C1R, HV315, APOL1, F13A, FIBA, FINC, TSP1, ACTB	(117)		Cell mediated rejection (CMR)	Isolated from 20mL or 500µL urines by differential centrifugation	Actin filament based process (47 proteins) Epithelial cell idfferenciation (21 proteins) Cytoskeleton organization (63 proteins) HPX, TSPAN, LGALS3BP		(110, 112)
	Antibody Mediated Rejection (ABMR)	Isolated from plasma by differential centrifugation	KV302, HV304	FIBB, FIBG, C1QA, C1R, HV315, ITIH1, APOL1, F13A, FIBA, FINC, TSP1, ACTB	(117)		AntiBody Mediated Rejection (ABMR)	isolated from 200µL plasma with exoRNeasy Serum/Plasma Midi Kit	GP130, CAV1, DARC, SH2D1B, CCL4, TNFα,	IL-10, IL-23α	(138)
	Cardiac Allograft Vasculopathy	Isolated from BAL fluids and serum by ultracentrifugation	Myo, Vim		(140)		AntiBody Mediated Rejection (ABMR)	Isolated from 50mL pool of urines by differential centrifugation	Protein transport (97 proteins) Protein localization (103 proteins) Acute inflammatory response (18 proteins) Proteine maturation by peptide bond cleveage (15 proteins0 APOA1, PIGR, TTR, AZGP1, HPX and CP)		(110, 111)
Islets	Acute rejection	Isolated from plasma using Invitrogen Total Exosome Isolation Kit from serum	Hemopexin, C3, 39 miRNAs	HSC70, angiopoietin-1	(117, 118)		Acute rejection	Isolated from 10mL urines by differential centrifugation	A2M, APOA2, APOM, CD5L, CLCA1, FGA, FGB, IGHM, DEFA5, PROS1 and KIAA0753		(113)

Different studies have linked graft rejection with differential expression of various proteins and miRNA. This table synthetize the markers found in the literature separated by transplanted organ, isolation method, mode of rejection and the compartment where the EV were harvested from. To note, an important part of these markers has been found in the animal model and all of them have not been assessed in human yet.

On the other hand, EVs are extensively studied for their utilization as therapeutics in transplantation. EVs derived from regulatory cells have shown immunosuppressive abilities that are not identical but complementary to their parental functions. EV therapies are based on the same principles as cellular therapies and mostly involve common pathways. However, EV therapies are free of any cellular context and EVs have no replicative properties. There is no risk of engrafting *in vitro* engineered cells in patients. However, no data on the long-term impact of EV-induced immune suppression are available yet in transplanted patients. We reviewed *in vivo* studies that have demonstrated the efficacy of such treatments in delaying graft rejection in preclinical models. The results from one patient suffering from GvHD and treated with MSC EVs were encouraging (75), but we are still far from clinical trials for solid organ transplantation or GvHD as we still need GMP suitable protocols for EVs preparation (e.g. EVs origin, isolation method, monitoring). Initiatives emerge to propose precise recommendations about EVs handling (144) and are taken to grow. As far as we know, the choice of the isolation methods depends from the attempted perspectives since different protocols may alter different EV cargo compartment. Among the 3 main components of EVs, namely lipids, proteins and RNA, the researches in the field of transplantation mainly focuses on miRNA-induced gene inhibition and protein interactions with relatively important knowledge in the search of biomarkers or immunomodulators, whereas the impact of lipids has not been extensively studied, whilst they have demonstrated their role in immune functions (39, 40, 43, 44, 48).

## Author Contributions

NS and MU wrote the original manuscript. AD, AS, FG, HM, and SB revised the manuscript and actively contributed to the final version. All authors contributed to the article and approved the submitted version.

## Funding

The editing and publishing fees were financially supported by Ceva Santé Animale CIFRE grant n° 2019/0388 and by the Institut de Recherche en Santé Respiratoire des Pays de la Loire (IRSRPL).

## Conflict of Interest

The authors declare that the research was conducted in the absence of any commercial or financial relationships that could be construed as a potential conflict of interest.

## Publisher’s Note

All claims expressed in this article are solely those of the authors and do not necessarily represent those of their affiliated organizations, or those of the publisher, the editors and the reviewers. Any product that may be evaluated in this article, or claim that may be made by its manufacturer, is not guaranteed or endorsed by the publisher.
